# Acute Myeloid Leukemia Presenting As Thrombotic Thrombocytopenic Purpura

**DOI:** 10.7759/cureus.6869

**Published:** 2020-02-04

**Authors:** Michael P Kucharik, David Waldburg, Anitha Chandran, Alison Kohn, Roozbeh Nazarian

**Affiliations:** 1 Internal Medicine, Florida Atlantic University Charles E. Schmidt College of Medicine, Boca Raton, USA; 2 Internal Medicine, Boca Raton Regional Hospital, Boca Raton, USA

**Keywords:** thrombotic thrombocytopenic purpura, acute myeloid leukemia, microangiopathic hemolytic anemia, ttp, aml, myelodysplastic, thrombocytopenia

## Abstract

We present a case of acute myeloid leukemia (AML) with myelodysplasia-related changes that presented as thrombotic thrombocytopenic purpura (TTP). Our patient presented with the classic pentad of TTP symptoms: anemia, thrombocytopenia, fever, elevated creatinine, and altered mental status. After a failure to respond to plasmapheresis therapy, we proceeded with a bone marrow biopsy and fluorescent in situ hybridization, which supported formal diagnosis of AML with myelodysplasia-related changes. Our case is an extremely rare presentation of a rare condition, as there have been no reported cases of AML with myelodysplasia-related changes presenting as TTP.

## Introduction

Acute myeloid leukemia (AML) is a type of cancer in which the bone marrow makes abnormal myeloblasts, red blood cells, or platelets. Proliferation of abnormal cells in the bone marrow interferes with normal production of normal blood cells [1]. Classically, patients present with lethargy, easy bleeding and bruising, and increased risk of infection. Thrombotic thrombocytopenic purpura (TTP) is a rare blood disorder caused by antibodies against the enzyme ADAMTS13, which results in the fragmentation of platelets by uncleaved von Willebrand multimers and subsequent formation of blood clots in microvasculature throughout the body. Classically, these patients present with low-grade fevers, anemia, thrombocytopenia, acute kidney injury, altered mental status, and schistocytes on peripheral blood smear [2]. We present a patient whose clinical presentation aligned with TTP, but upon further investigation, was found to have a rare form of AML.

## Case presentation

This is a 75-year-old male who presented to the emergency department with altered mental status, non-bloody diarrhea, subjective fevers, and intermittent epistaxis, each of which had been present for the past two weeks. Past medical history was notable for non-insulin-dependent diabetes mellitus, paroxysmal atrial fibrillation, anticoagulated with apixaban 5 mg twice daily, and hypertension. The patient's vital signs were notable for a temperature of 98.6°F, a heart rate of 98 bpm, a blood pressure of 110/78 mmHg, a respiratory rate of 18, and an oxygen saturation of 96%. At this time, physical exam was notable for a lethargic, obese male in no acute distress, epistaxis from right nostril, 3/6 crescendo-decrescendo murmur in the right upper sternal border, hepatosplenomegaly, and petechiae scattered across the patient’s bilateral lower extremities.

**Table 1:** Patient's Laboratory Values on Presentation

Parameter (units)	Value
Hemoglobin (g/dL)	9.2
Mean corpuscular volume (fL)	76
White blood cells (/µL)	8,500
Platelets (/µL)	11,000
Creatinine (mg/dL)	1.5
Prothrombin time (seconds)	17.1
Partial thromboplastin time (seconds)	36.5
D-Dimer (µg/mL)	1.67
Fibrinogen (mg/dL)	630
Lactic acid dehydrogenase (units/L)	605
Haptoglobin (mg/dL)	243
Reticulocyte count (cells/µL)	0.008

The patient's creatinine was increased from a baseline of 1.1, which was recorded one month prior to presentation. The complete blood count was re-ordered following a transfusion of one unit of platelets, which was notable for a decrease in platelets from 11,000 per µL to 9,000 per µL.

**Figure 1:**
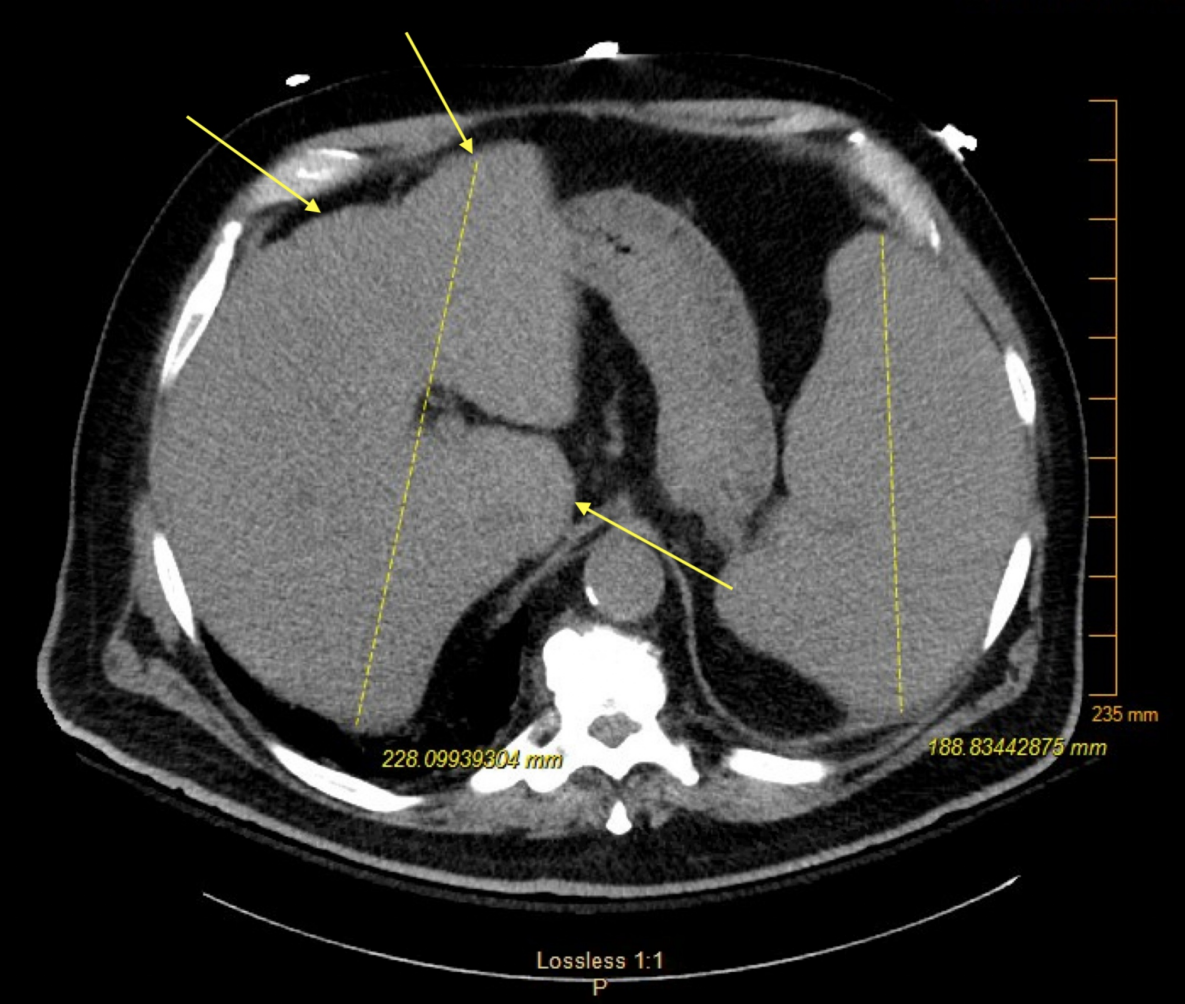
Hepatosplenomegaly with few non-specific hepatic nodules.

Non-contrast computerized tomography (CT) of the patient’s abdomen featured hepatosplenomegaly (Figure 1). At this time, a peripheral blood smear was ordered, which was remarkable for 2% schistocytes without blasts. The patient underwent plasmapheresis treatment for suspected TTP for a total of five days. The patient did not respond to therapy, as his platelets continued to oscillate between 8,000 and 12,000 µL.

After the plasmapheresis was completed, the decision was made to proceed with a bone marrow biopsy and fluorescent in situ hybridization, which was remarkable for a long arm deletion on chromosome 5, which supported a formal diagnosis of AML with myelodysplasia-related changes.

Throughout the entire hospital course, the patient was given 11 units of packed red blood cells and 12 units of platelets for sustained anemia and thrombocytopenia, respectively. Following consultation with hematology, due to the patient’s age and comorbidities, the decision was made to treat him with a five-day course of decitabine, a low-intensity chemotherapy drug. Following consultation with nephrology, the patient was placed on dialysis for the duration of chemotherapy for tumor lysis syndrome prophylaxis. On day 13 of his hospitalization, the patient and family elected for supportive care. On the morning of day 14, the patient was pronounced dead due to complications from AML and subsequent treatment.

## Discussion

Although AML with myelodysplastic changes is a common subset of acute leukemias, this case is particularly rare because of the patient's presentation. Since the patient presented with the classic pentad of TTP symptoms (fever, anemia, thrombocytopenia, acute kidney injury, and altered mental status) and 2% schistocytes were seen on peripheral blood smear, diagnosis of AML was consequently delayed.

Even though anemia, thrombocytopenia, and fever are characteristic symptoms of leukemia, it is rare for AML to present with acute kidney injury, acute change in mental status, and decreasing platelets following platelet transfusion [1]. This is the first reported case of AML with myelodysplastic changes that presented with the classic pentad of TTP symptoms, schistocytes on peripheral smear, and absence of myeloblasts on peripheral smear.

Relying on the classic presentation of TTP symptoms is unreliable, as only 20%-30% of patients present with the pentad [3]. Moreover, the presence of schistocytes is not specific for TTP. In fact, in TTP, schistocytes typically range between 3% and 10% [4]. Schistocytes may also be seen in disseminated intravascular coagulation (DIC), hemolytic uremic syndrome (HUS), and malfunctioning heart valves [4].

In our patient, increased haptoglobin, increased fibrinogen, and absence of bloody diarrhea made TTP, DIC, and HUS less likely, respectively. Moreover, the severely low reticulocyte count made malignancy or aplastic anemia much more likely. However, since the patient's platelets remained <15,000/µL throughout his hospitalization, a bone marrow biopsy to confirm a diagnosis of acute leukemia was delayed. Without a formal diagnosis, we elected for plasmapheresis exchange transfusion since it is recommended in any patient with suspected TTP due to mortality as high as 90% without treatment [2].

Although AML is a relative rare disease, it is the most common acute leukemia in adults [5]. Non-Hispanic white males have a higher incidence than other racial and ethnic groups and the median age at diagnosis is 65 years of age [5].

The etiologies of underlying chromosomal abnormalities in most cases of AML are largely unknown [6]. The WHO 2008 classification of AML categorizes the condition into subtypes, which aids in diagnosis and prognosis [7]. Our patient was found to have a 5q deletion, which most often occurs in elderly patients with prior myelodysplastic syndrome and is associated with rapid deterioration and poor prognosis [8]. Our patient’s prognosis was especially poor considering he had possible metastatic lesions in his liver and spleen [8].

Treatment of AML is largely based on the patient’s age, comorbidities, current functional status, and goals of treatment at time of diagnosis, as treatment is highly variable and may actually decrease life expectancy [9]. In patients with AML with deletions of chromosome 5q, there is insufficient data to predict the possibility of complete remission in elderly patients with comorbidities [10]. However, delaying treatment has been associated with a decreased rate of complete remission and decreased life expectancy by three to five months in select patient populations [11,12].

## Conclusions

There have been no reported cases of AML with myelodysplasia-related changes presenting as TTP. Early diagnosis of AML in elderly patients is especially important due to the rapid progression of this disease. In summation, this case shows that AML should be under consideration in elderly patients presenting with unexplained cytopenias and constitutional symptoms, even in the absence of blasts on peripheral blood smear. It is important to diagnose AML in its early stages and manage it appropriately in order to prolong survival and increase the chances of complete remission. This case highlights the importance of a high clinical index of suspicion for AML in patients who present with unexplained cytopenias and constitutional symptoms even when there are distracting symptoms and an absence of blasts on peripheral smear.
